# Editors’ Note

**DOI:** 10.5195/ijt.2017.6239

**Published:** 2017-11-20

**Authors:** ELLEN R. COHN, JANA CASON

## ISSUE OVERVIEW

The current issue of the International Journal of Telerehabilitation (IJT) contains original research, policy, and a country report (India). A letter to the editor cogently describes the challenges faced by occupational audiologists in the United States, due to the current absence of interstate professional license portability. There is no doubt that the lead article, “User

Authentication in Mobile Phones and Its Application to Healthcare,” is highly relevant given the current security challenged environment.

What do these articles have in common? They collectively describe the current status of telerehabilitation: successes, challenges, and future needs.

## ON (PERSISTENT) GRATITUDE

In this season of giving thanks, we recognize that the IJT could not be produced without the generous efforts of others. We are grateful to new and returning reviewers; Sections Editor, William E. Janes, OTD, MSCI, OTR/L; colleagues at the Rehabilitation Engineering Research Center on Information and Communication Technology Access at the University of Pittsburgh; and, our publishers, Timothy S. Deliyannides, Director, Office of Scholarly Communication and Publishing, and Head, Information Technology, University Library System, and Vanessa Gabler and Michelle Bradbury**,** Electronic Publications Associates at the University of Pittsburgh.

Did you know recent evidence suggests that expressing gratitude on a daily basis (i.e., by daily recording three items for which a person is thankful for at least six months), can benefit physical and psychological health? Yet another reason to give a huge “tele-thanks” to all those who are advancing the status of telerehabilitation.

## CALL FOR SUBMISIONS

The next volume of the International Journal of Telerehabilitation will be published in Spring, 2018. We cordially invite your 2018 submissions by mid-January 2018, and accept original research, case studies, viewpoints, technology reviews, book reviews, and country reports that detail the current status of telerehabilitation.

Sincerely,

Ellen R. Cohn, PhD, CCC-SLP, ASHA-F

IJT Editor

Jana Cason, DHS, OTR/L, FAOTA

Senior Associate Editor

